# Supplementary material for: Methylphenidate reorganizes cortical hierarchy through dopaminergic modulation
Source: Nat Commun. 2025 Dec 13;17:791. doi: 10.1038/s41467-025-67477-y (PMC12824357; doi:10.1038/s41467-025-67477-y)
Supplement: Supplementary file 1 — Supplementary Information [file 41467_2025_67477_MOESM1_ESM.pdf]

Supplementary data

Methylphenidate reorganizes cortical hierarchy through dopaminergic modulation

Dardo Tomasi[1], Peter Manza[1,2], Şükrü Barış Demiral[1], Weizheng Yan[1], Kylee B. Miller[1], Faith Veenker[1], Joshua Zhao[1], Christina Lildharrie[1], Michele-Vera Yonga[1], Sarah Abey[1], Michaelene VanDine[1], Gene-Jack Wang[1], Nora D. Volkow[1]

[1]National Institute on Alcohol Abuse and Alcoholism, National Institutes of Health, Bethesda, Maryland, USA.

[2]Kahlert Institute for Addiction Medicine, University of Maryland, Baltimore, Maryland, USA.

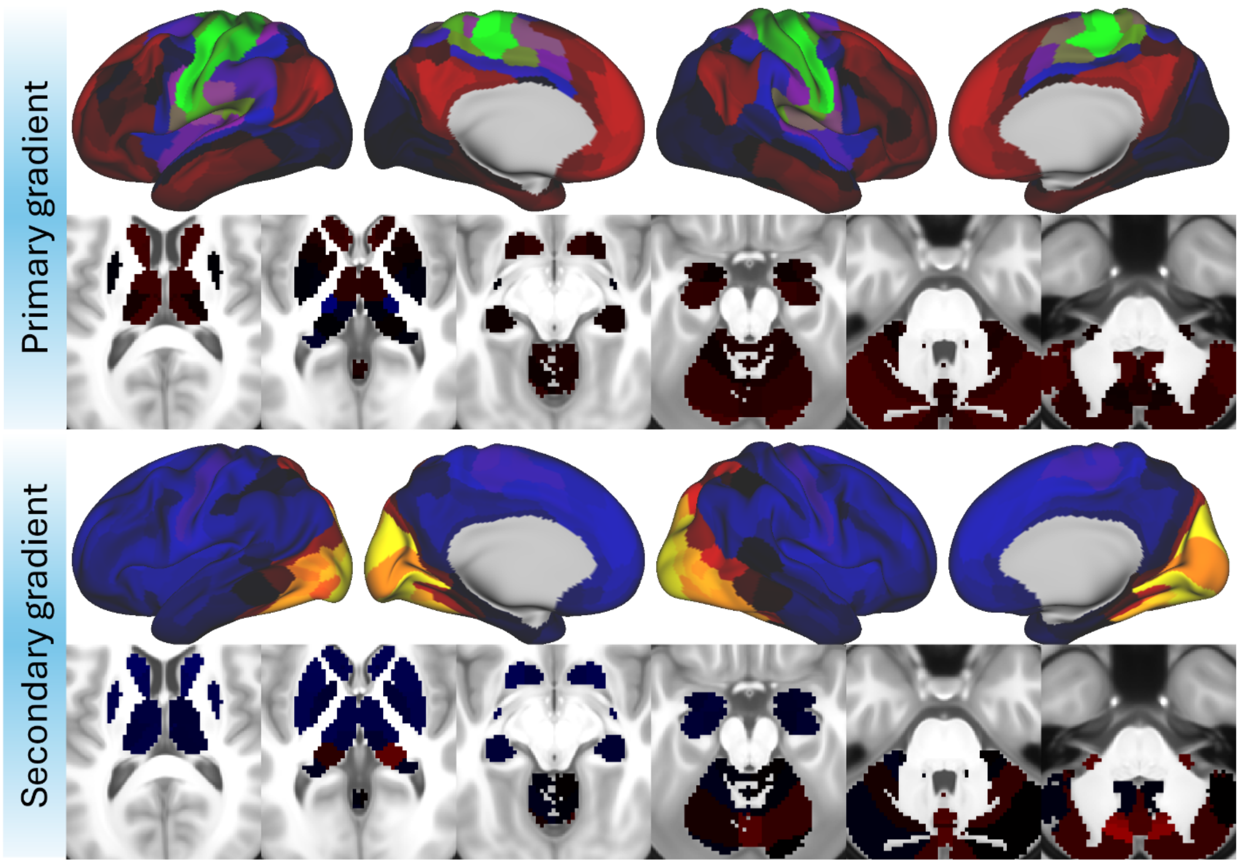

Figure S1: Templates for the low-dimensional gradients. Group-level templates for the primary and secondary gradients were computed from the average parcellated connectome.

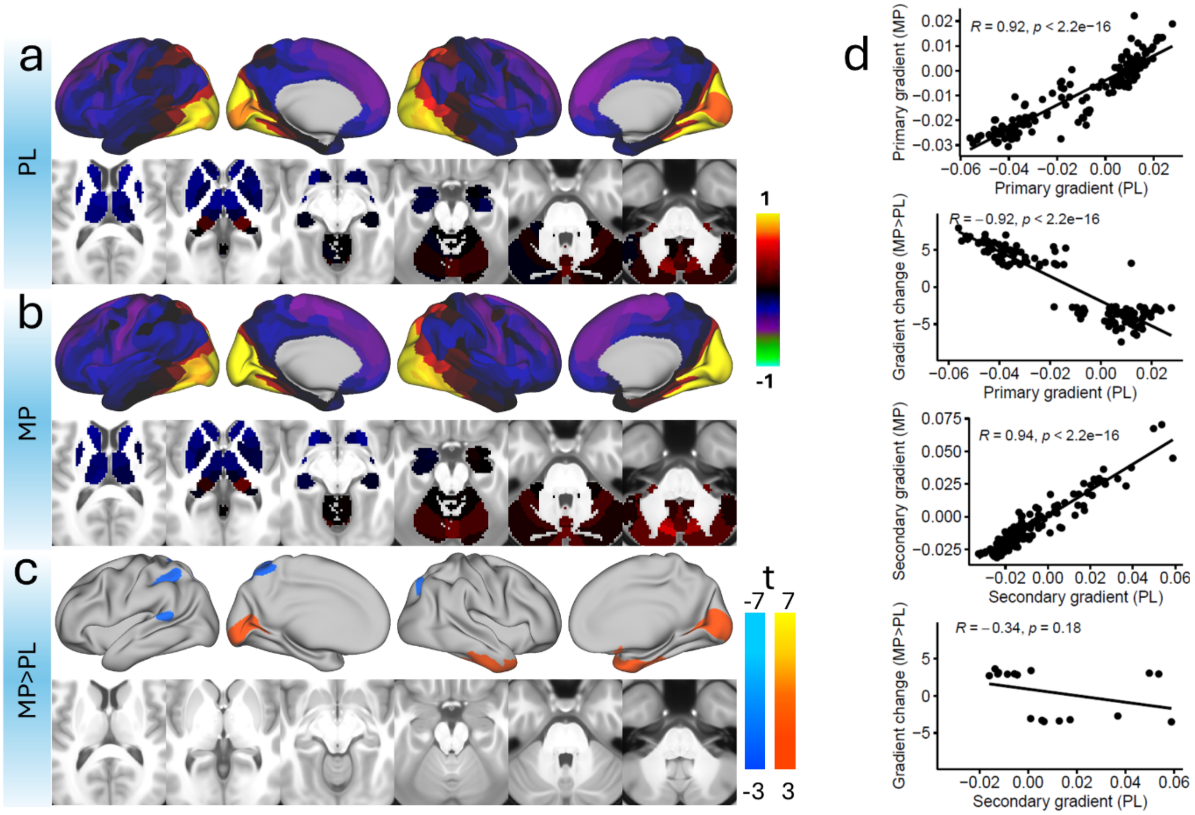

Figure S2: Effect of MP on the secondary gradient of brain functional organization. (**a**, **b**) Surface renderings display the strength of the secondary gradient overlaid on lateral and medial views of the left and right hemispheres, along with six axial slices covering subcortical regions, for the placebo (PL; a) and methylphenidate (MP; b) conditions in 38 healthy adults. The parcellation atlas included 438 cortical and subcortical regions. (**c**) Statistical difference maps (t-scores) illustrate regions with significant changes in gradient strength between PL and MP, estimated using a linear mixed-effects (LME) model with age, sex, race, body mass index, and intelligence as covariates. The statistical maps are displayed using a false discovery rate (FDR) threshold of $p < 0.05$. (**d**) Scatter plots showing strong linear associations between PL and MP conditions for each gradient and between the strength of the primary gradient and the statistical significance in primary gradient changes induced by MP across 438 brain regions (compared to the lack of a similar association for the secondary gradient;

bottom).

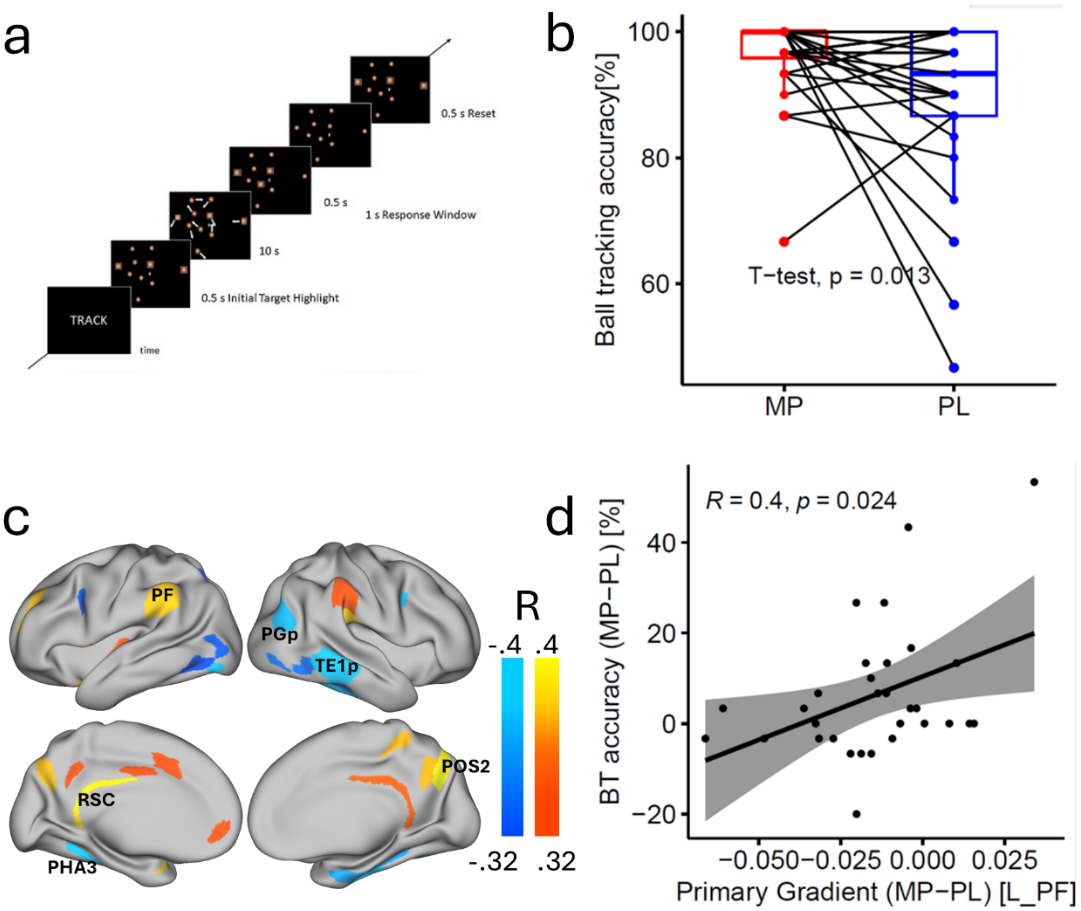

Figure S3: Effects of methylphenidate (MP) on visual attention (VA) accuracy and its association with changes in the principal gradient. (**a**) Schematic of the non-verbal visual attention task performed in the MRI scanner. Participants covertly tracked two or three target balls among ten moving balls while maintaining central fixation. After each trial, they indicated via button press whether a new set of highlighted balls matched the original targets. Feedback was provided to reorient attention before the next trial. (**b**) Paired plot illustrating significantly higher average VA accuracy in the MP condition compared to placebo (PL) across 32 healthy participants. Surface renderings on lateral and medial views of the left and right hemispheres (**c**) showing the spatial distribution of correlations between MP-induced changes in VA accuracy and changes in the strength of the principal gradient using an uncorrected threshold $P<0.05$. (**d**) Scatter plot illustrating the positive association between individual MP-related changes in VA accuracy and changes in the strength of the principal gradient in the left PF parcel of the inferior parietal cortex. Shaded area denotes 95% confidence intervals. PF: supramarginal gyrus; RSC: retrosplenial cortex; POS2: parieto-occipital sulcus area 2; PHA3: peri-hippocampal area 3; TE1p: temporal area 1 posterior; PGp: posterior angular gyrus.

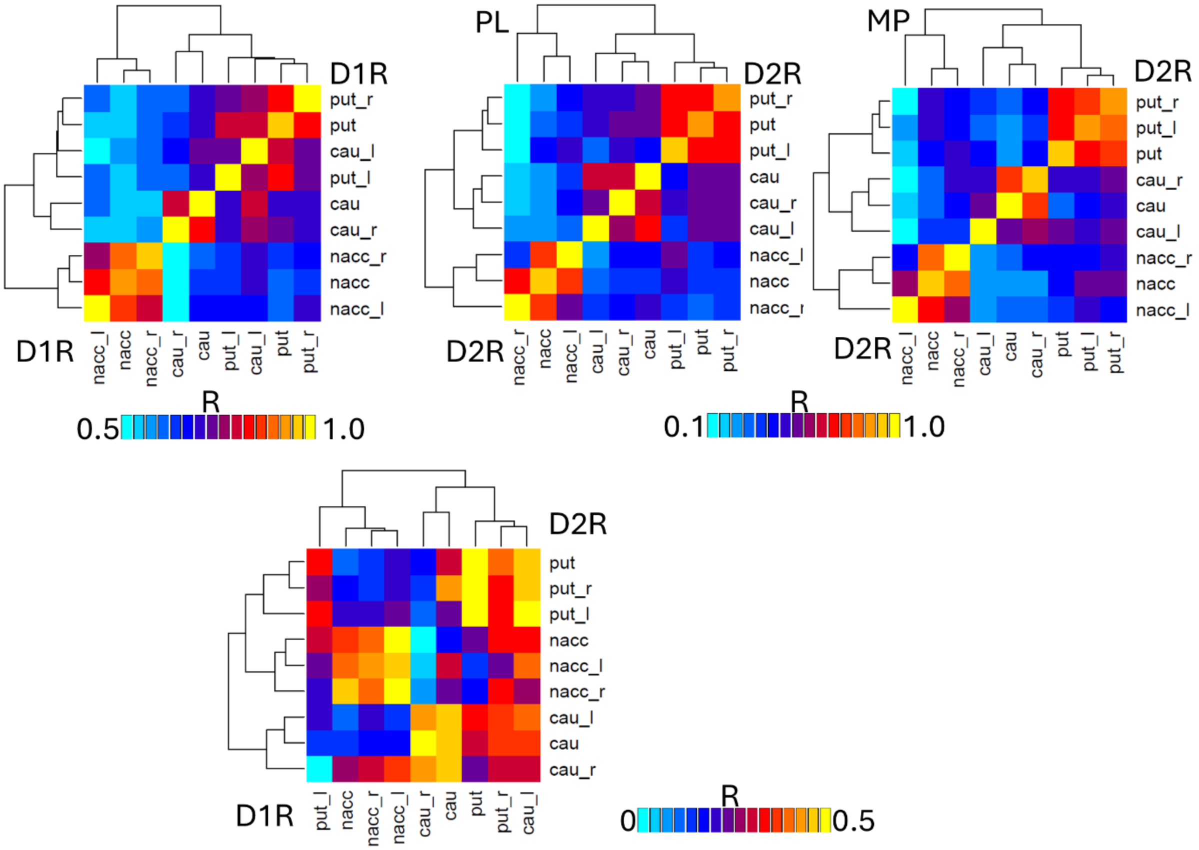

Figure S4: Correlations between non-displaceable binding potentials corresponding to D1R availability (left matrix) and D2R availability (middle and right matrices) across striatal regions (averaged in left, right, and bilateral putamen, put, caudate, cau, and nucleus accumbens, nacc). The bottom matrix shows the correlations between D1R and D2R availability across striatal regions. PL: placebo; MP: methylphenidate. 38 healthy adults.

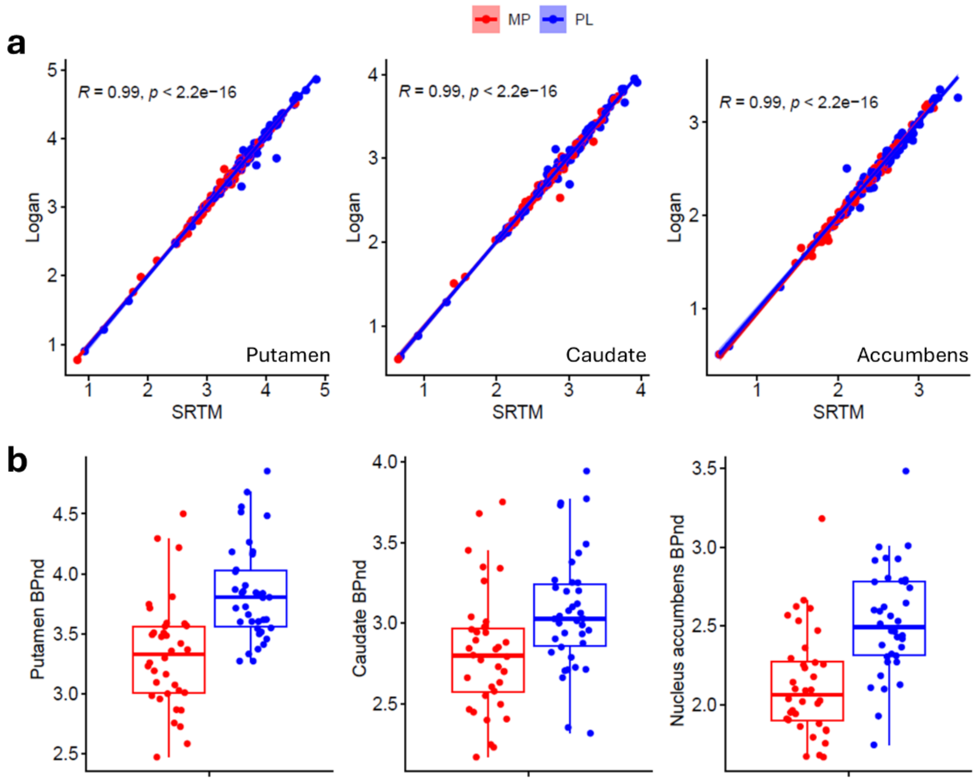

Figure S5: (**a**) Scatter plot showing the linear relationship between BPnd values for raclopride binding estimated using the Simplified Reference Tissue Model (SRTM) and Logan graphical analysis across placebo (PL) and methylphenidate (MP) conditions. Each point represents an individual. (**b**) Box plots showing the distribution of BPnd values for raclopride binding for PL and MP.

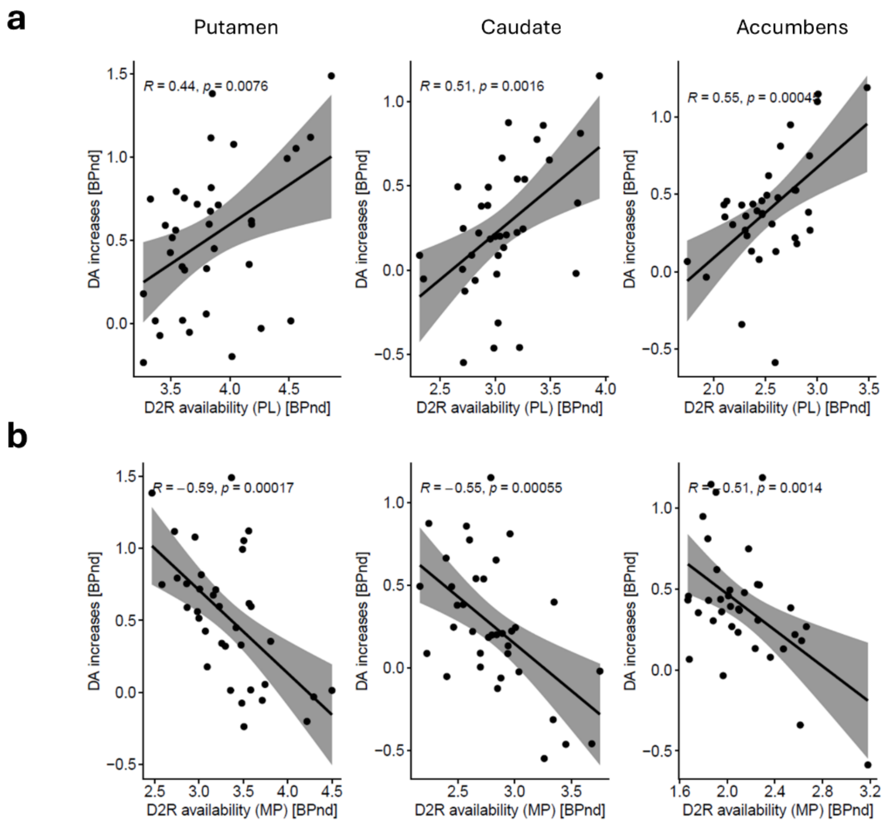

Figure S6: Scatter plots showing linear associations between DA increases and D2 receptor availability in the striatum for placebo (**a**) and methylphenidate (**b**) conditions.

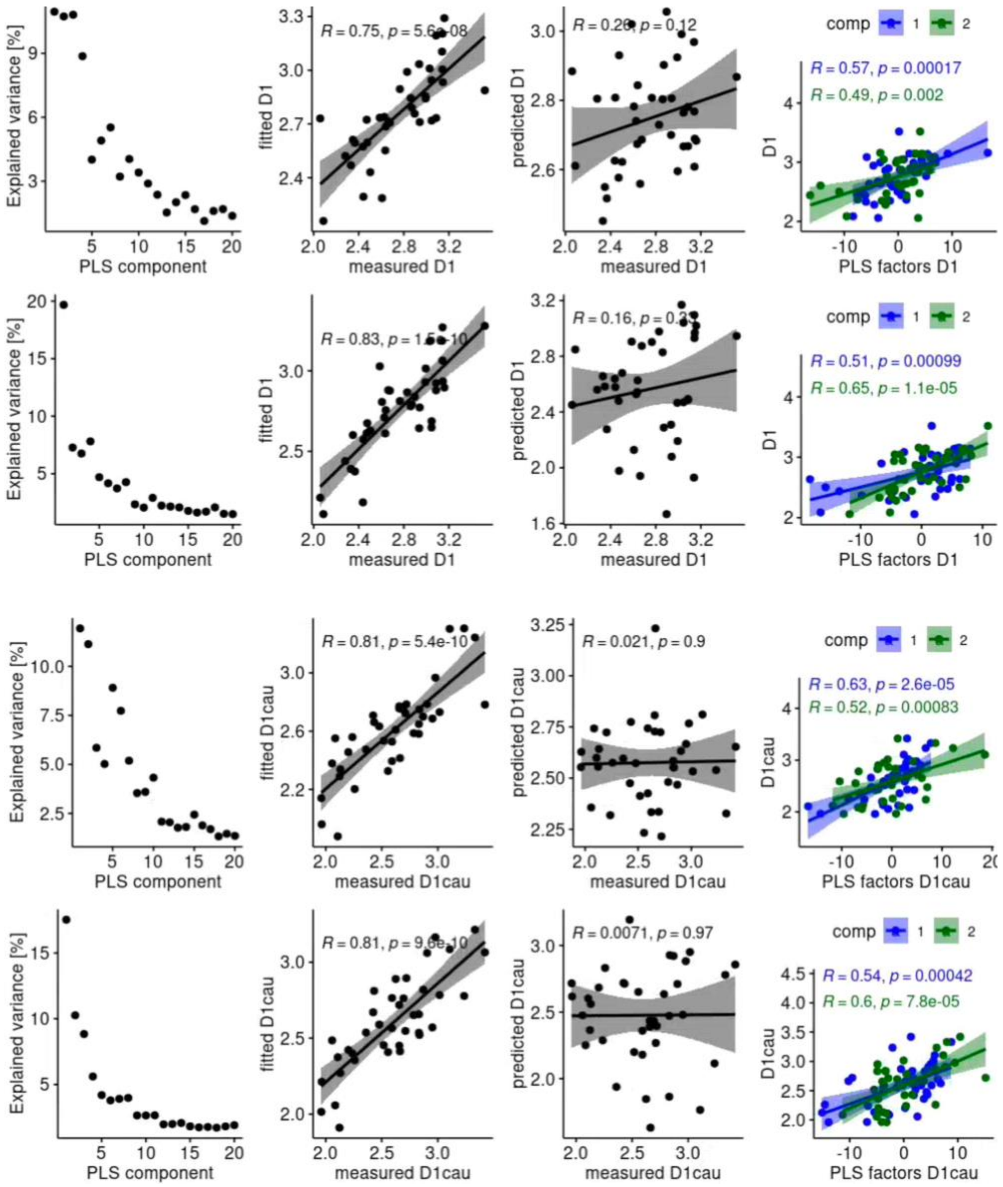

Figure S7: Partial least squares (PLS) regression predicting D1 receptor availability in the putamen (top) and caudate (bottom) from the primary gradient across 438 brain parcels. The top row includes: (i) explained variance by PLS components, (ii) fitted values from leave-one-out cross-validation (LOO-CV) versus measured values for the PL session, (iii) predicted D1 receptor availability in MP using the PLS model trained on PL, and (iv) the relationship between the measured metric and PLS factors for components 1 and 2. The bottom row shows the same metrics but with the model trained on MP and tested on PL.

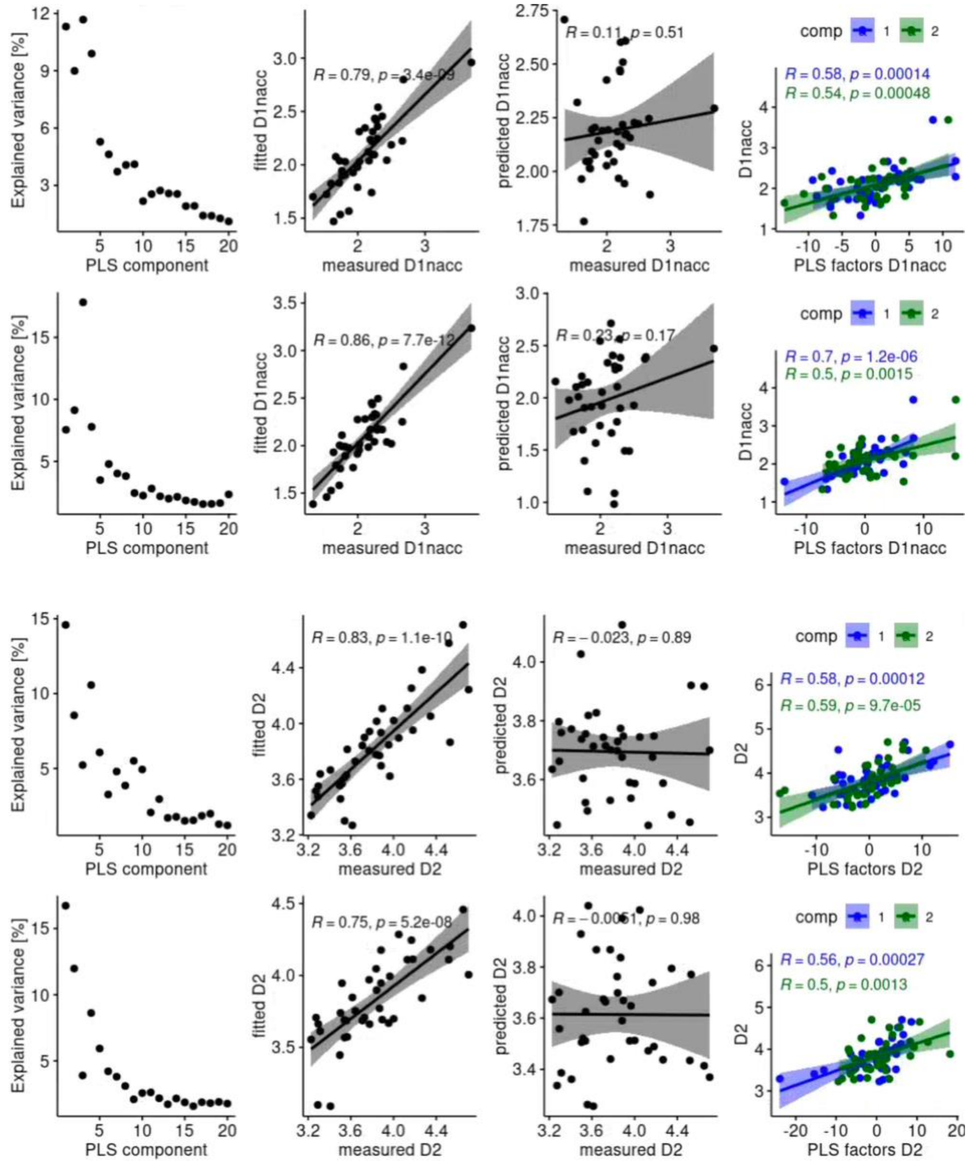

Figure S8: Partial least squares (PLS) regression predicting D1 receptor availability in the nucleus accumbens (top) and D2 receptor availability in the putamen (bottom) from the primary gradient across 438 brain parcels. The top row includes: (i) explained variance by PLS components, (ii) fitted values from leave-one-out cross-validation (LOO-CV) versus measured values for the PL session, (iii) predicted D1 receptor availability in MP using the PLS model trained on PL, and (iv) the relationship between the measured metric and PLS factors for components 1 and 2. The bottom row shows the same metrics but with the model trained on MP and tested on PL.

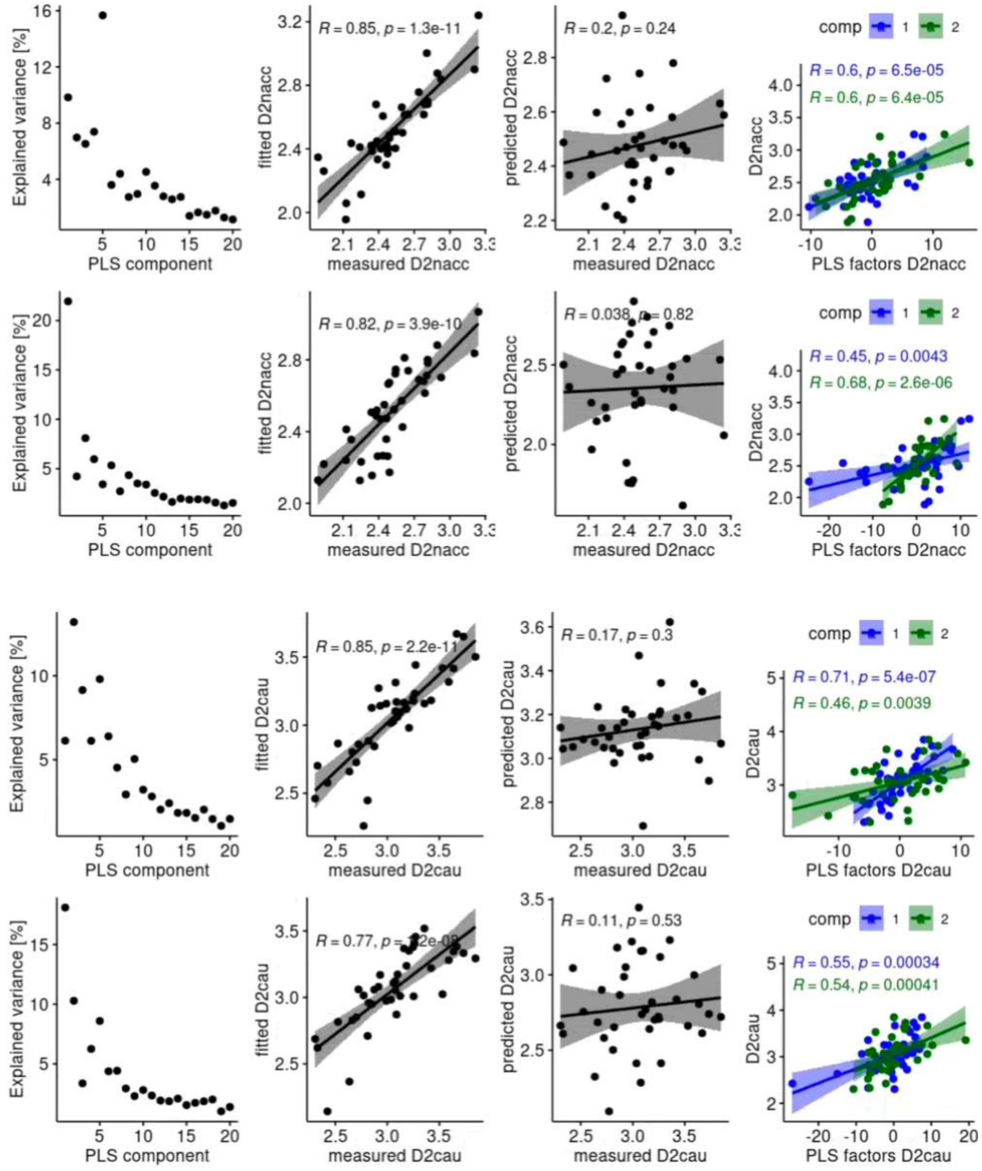

Figure S9: Partial least squares (PLS) regression predicting D2 receptor availability in the nucleus accumbens (top) and the caudate (bottom) from the primary gradient across 438 brain parcels. The top row includes: (i) explained variance by PLS components, (ii) fitted values from leave-one-out cross-validation (LOO-CV) versus measured values for the PL session, (iii) predicted D1 receptor availability in MP using the PLS model trained on PL, and (iv) the relationship between the measured metric and PLS factors for components 1 and 2. The bottom row shows the same metrics but with the model trained on MP and tested on PL.

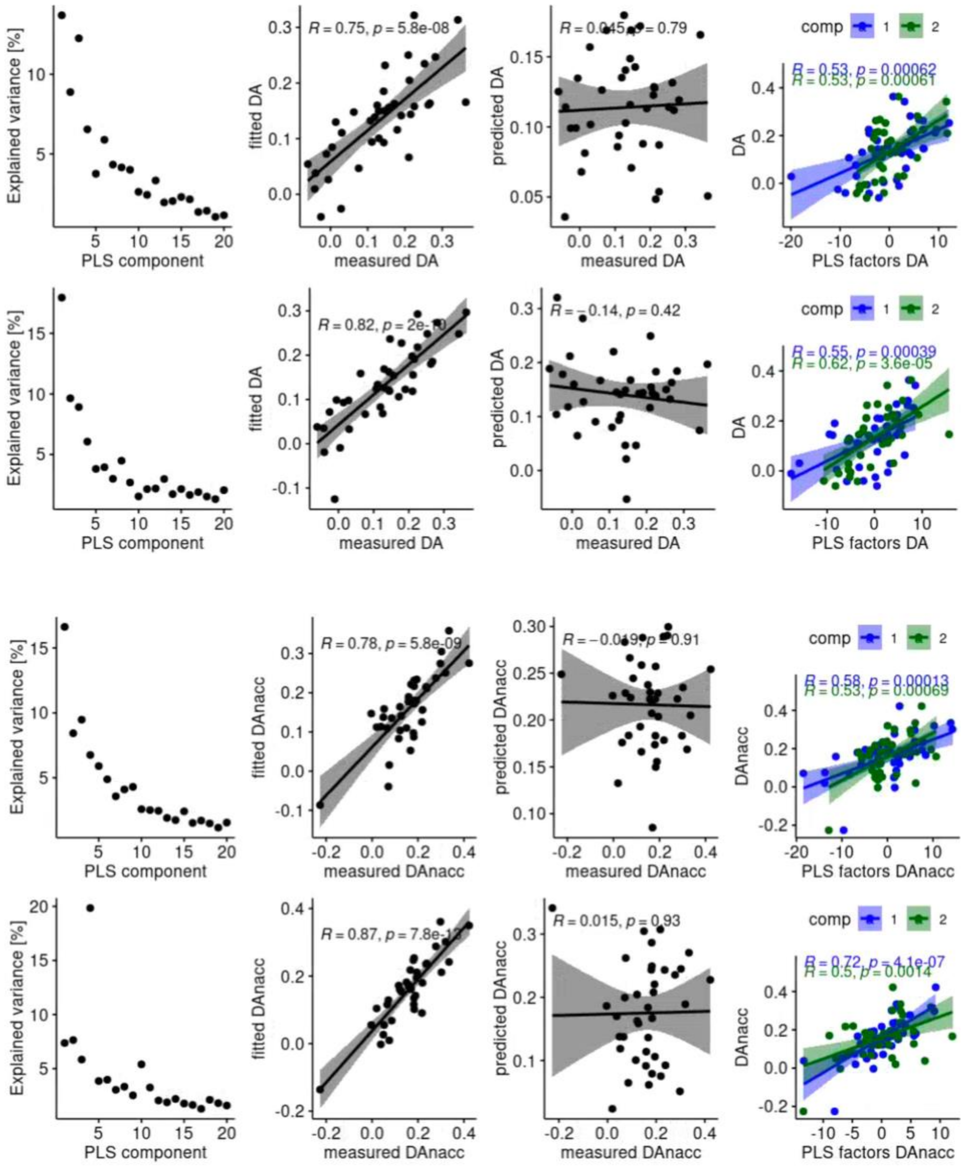

Figure S10: Partial least squares (PLS) regression predicting DA increases in the putamen (top) and nucleus accumbens (bottom) from the primary gradient across 438 brain parcels. The top row includes: (i) explained variance by PLS components, (ii) fitted values from leave-one-out cross-validation (LOO-CV) versus measured values for the PL session, (iii) predicted D1 receptor availability in MP using the PLS model trained on PL, and (iv) the relationship between the measured metric and PLS factors for components 1 and 2. The bottom row shows the same metrics but with the model trained on MP and tested on PL.

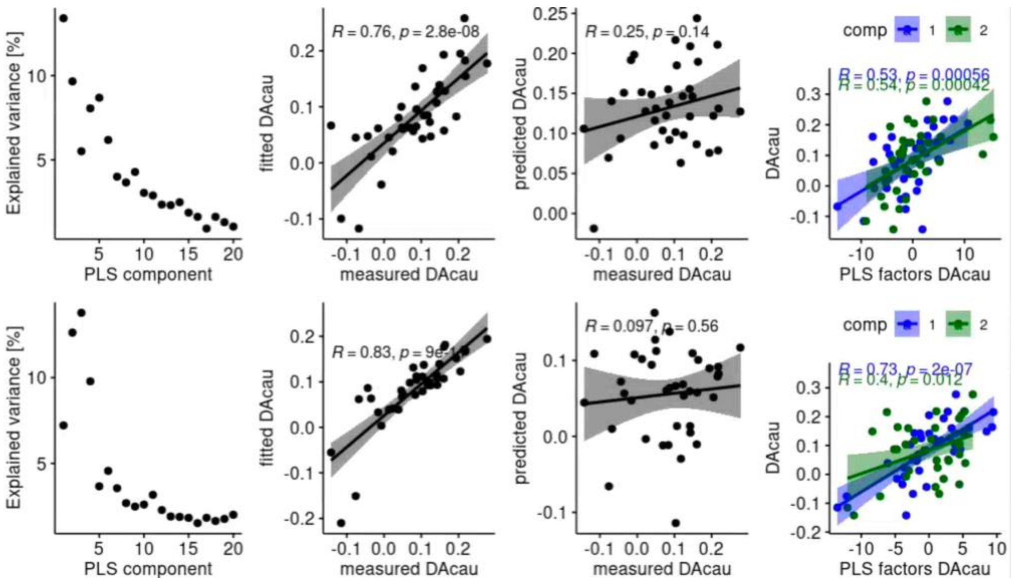

Figure S11: Partial least squares (PLS) regression predicting DA increases in the caudate from the primary gradient across 438 brain parcels. The top row includes: (i) explained variance by PLS components, (ii) fitted values from leave-one-out cross-validation (LOO-CV) versus measured values for the PL session, (iii) predicted D1 receptor availability in MP using the PLS model trained on PL, and (iv) the relationship between the measured metric and PLS factors for components 1 and 2. The bottom row shows the same metrics but with the model trained on MP and tested on PL.

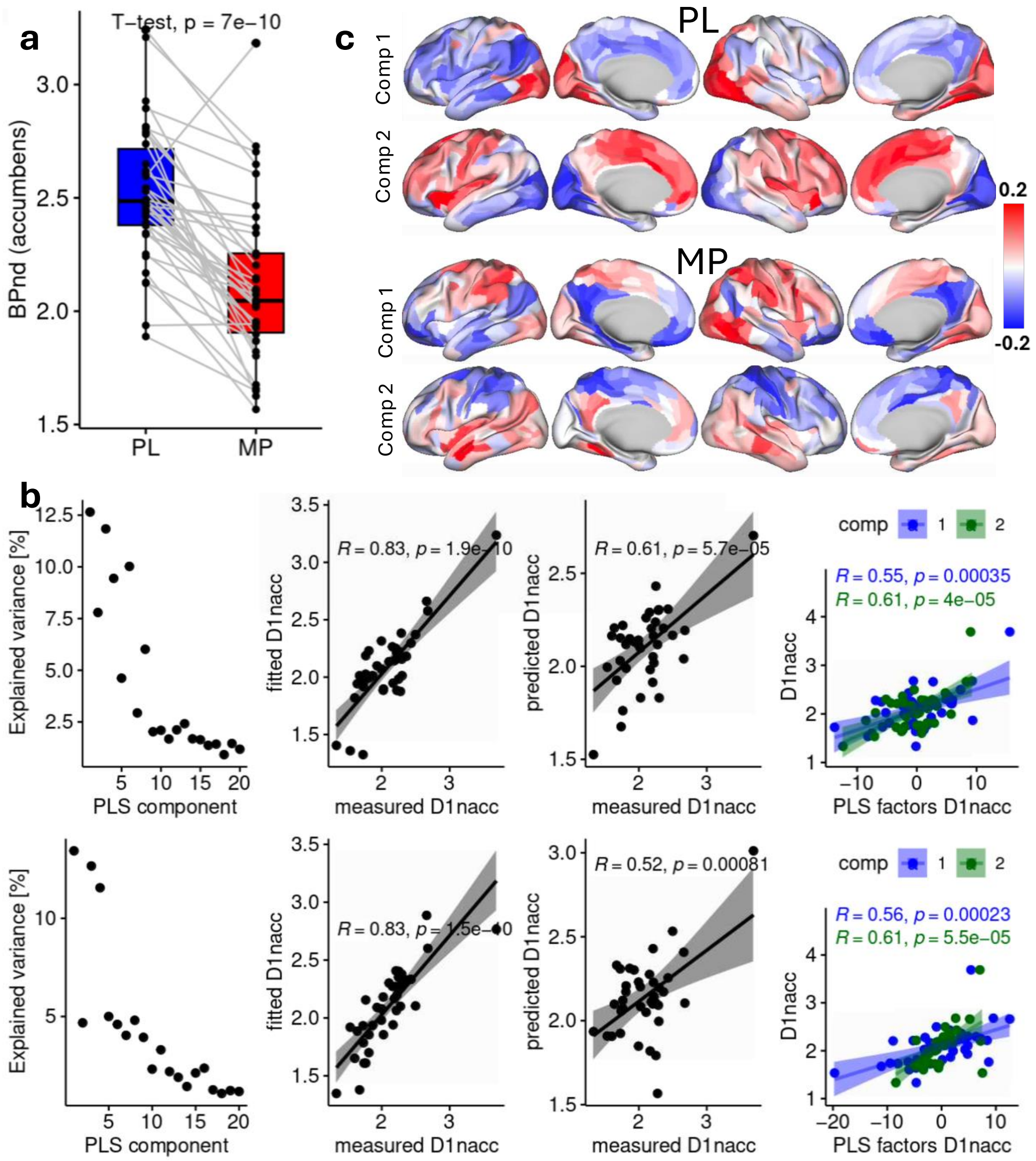

Figure S12: Dopamine receptor availability and PLS-based predictions in nucleus accumbens. (**a**) Paired plot showing the non-displaceable binding potential (BPnd) of [^{11}C]raclopride in nucleus accumbens for methylphenidate (MP) and placebo (PL) sessions across 38 healthy adults. (**b**) Partial least squares (PLS) regression predicting D1 receptor availability in the nucleus accumbens from the secondary gradient across 438 brain parcels with 2 components. The top row includes: (i) explained variance by PLS components, (ii) fitted values from leave-one-out cross-validation (LOO-CV) versus measured values for the PL session, (iii) predicted D1 receptor availability in MP using the PLS model trained on PL, and (iv) the relationship between the measured metric and PLS factors for components 1 and 2. The bottom row shows the same metrics but with the model trained on MP and tested on PL. **(c)** Loading patterns for components 1 and 2 of the PLS model predicting D1 receptor availability from the secondary gradient corresponding to MP and PL sessions. Surface renderings depict lateral and medial views, and axial slices highlight subcortical structures associated with the PLS components.

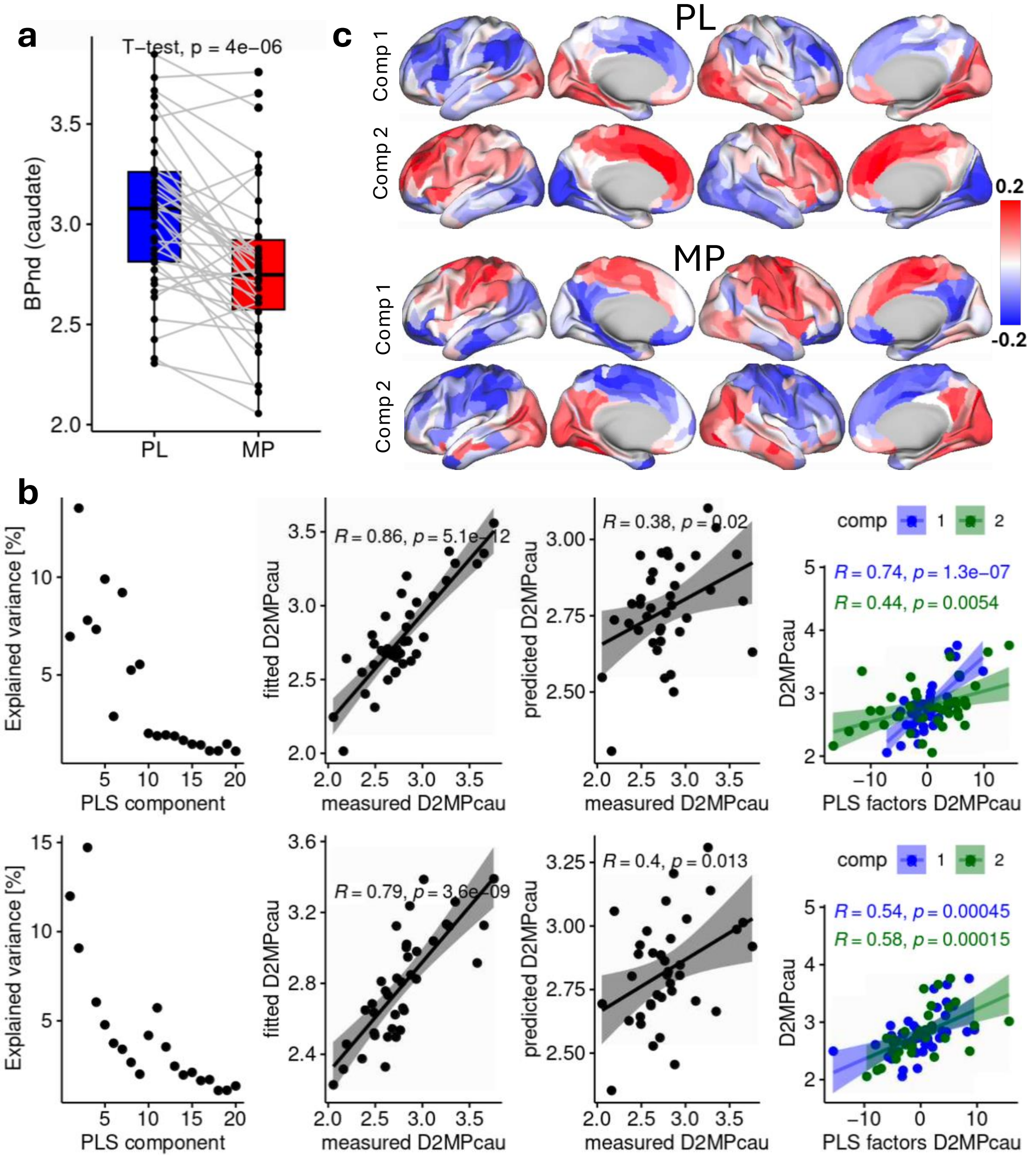

Figure S13: Dopamine receptor availability and PLS-based predictions in caudate. (**a**) Paired plot showing the non-displaceable binding potential (BPnd) of [^{11}C]raclopride in caudate for methylphenidate (MP) and placebo (PL) sessions across 38 healthy adults. (**b**) Partial least squares (PLS) regression predicting DA increases in caudate from the secondary gradient across 438 brain parcels with 2 components. The top row includes: (i) explained variance by PLS components, (ii) fitted values from leave-one-out cross-validation (LOO-CV) versus measured values for the PL session, (iii) predicted D1 receptor availability in MP using the PLS model trained on PL, and (iv) the relationship between the measured metric and PLS factors for components 1 and 2. The bottom row shows the same metrics but with the model trained on MP and tested on PL. **(c)** Loading patterns for components 1 and 2 of the PLS model predicting DA increases from the secondary gradient corresponding to MP and PL sessions. Surface renderings depict lateral and medial views, and axial slices highlight subcortical structures associated with the PLS components.

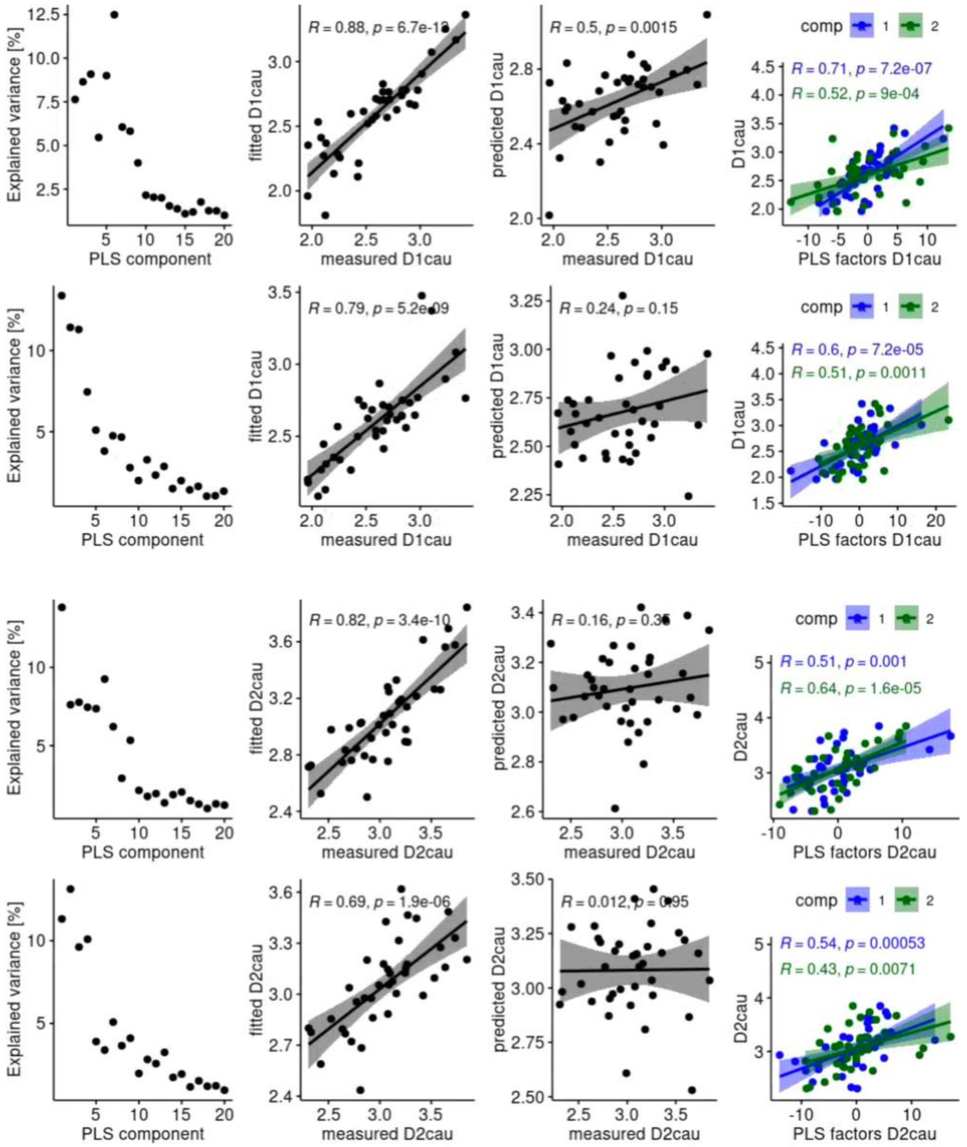

Figure S14: Partial least squares (PLS) regression predicting D1R (top) and D2R (bottom) in caudate from the secondary gradient across 438 brain parcels. The top row includes: (i) explained variance by PLS components, (ii) fitted values from leave-one-out cross-validation (LOO-CV) versus measured values for the PL session, (iii) predicted D1 receptor availability in MP using the PLS model trained on PL, and (iv) the relationship between the measured metric and PLS factors for components 1 and 2. The bottom row shows the same metrics but with the model trained on MP and tested on PL.

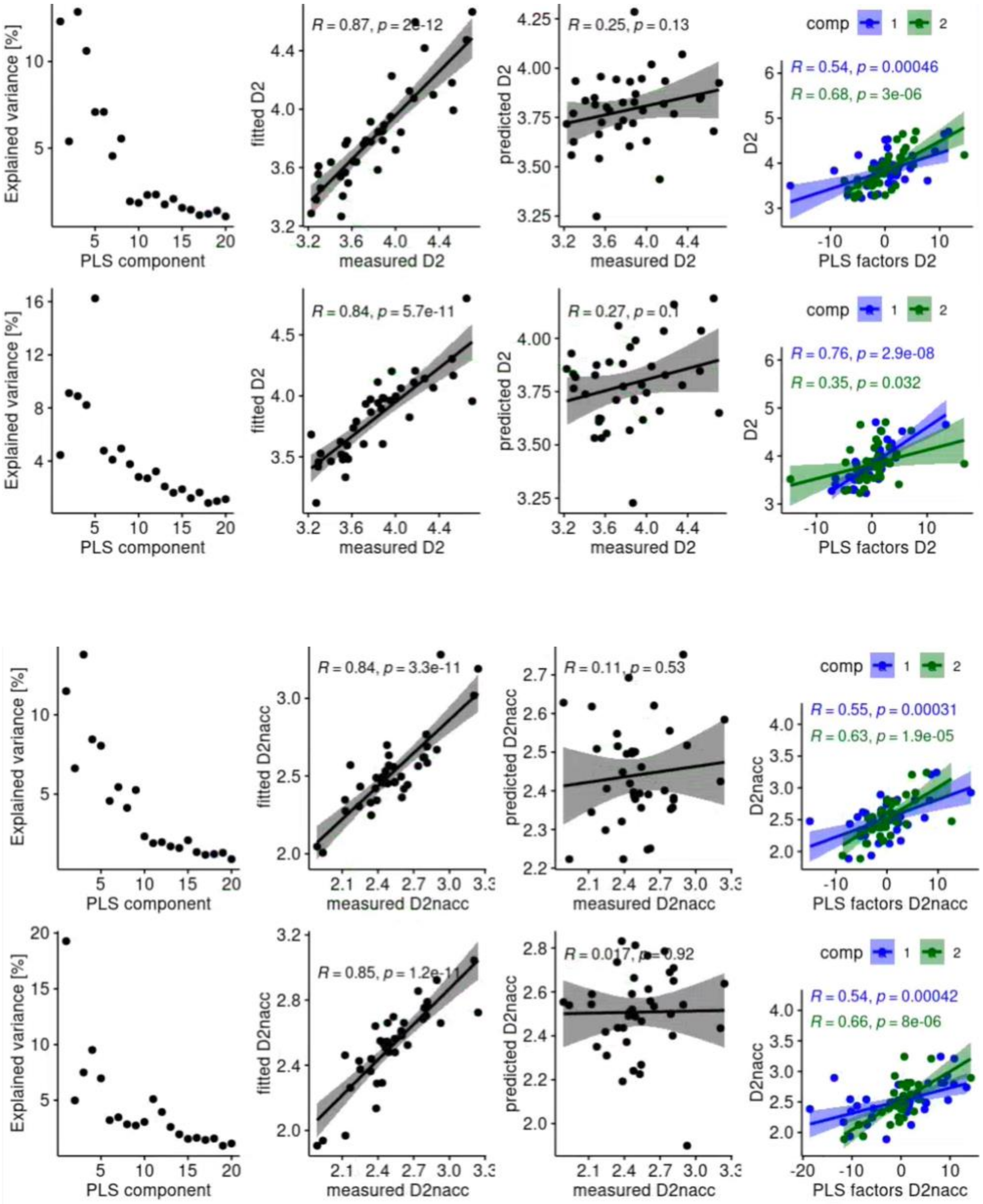

Figure S15: Partial least squares (PLS) regression predicting D2R in putamen (top) and nucleus accumbens (bottom) from the secondary gradient across 438 brain parcels. The top row includes: (i) explained variance by PLS components, (ii) fitted values from leave-one-out cross-validation (LOO-CV) versus measured values for the PL session, (iii) predicted D1 receptor availability in MP using the PLS model trained on PL, and (iv) the relationship between the measured metric and PLS factors for components 1 and 2. The bottom row shows the same metrics but with the model trained on MP and tested on PL.

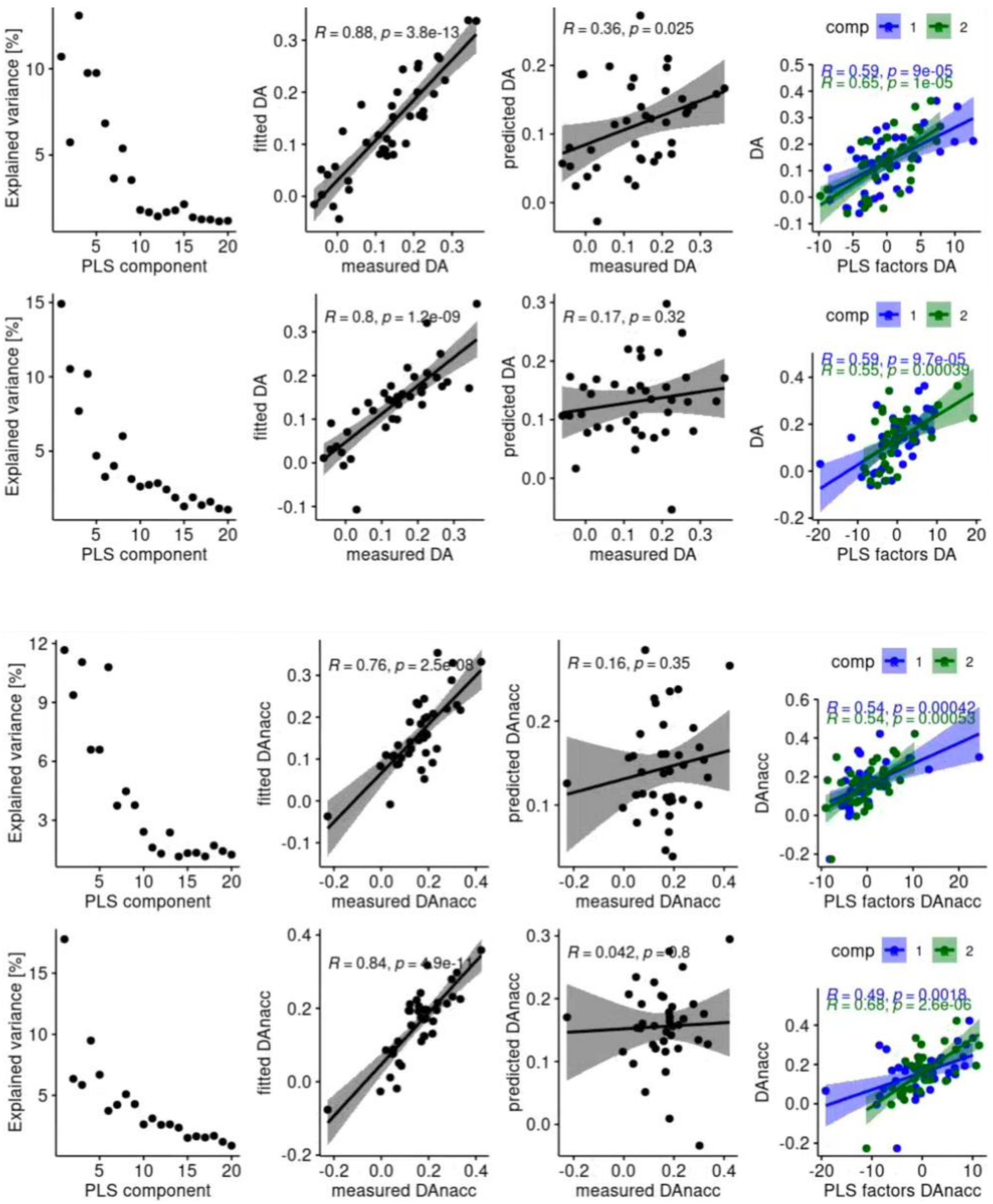

Figure S16: Partial least squares (PLS) regression predicting DA increases in putamen (top) and nucleus accumbens (bottom) from the secondary gradient across 438 brain parcels. The top row includes: (i) explained variance by PLS components, (ii) fitted values from leave-one-out cross-validation (LOO-CV) versus measured values for the PL session, (iii) predicted D1 receptor availability in MP using the PLS model trained on PL, and (iv) the relationship between the measured metric and PLS factors for components 1 and 2. The bottom row shows the same metrics but with the model trained on MP and tested on PL.

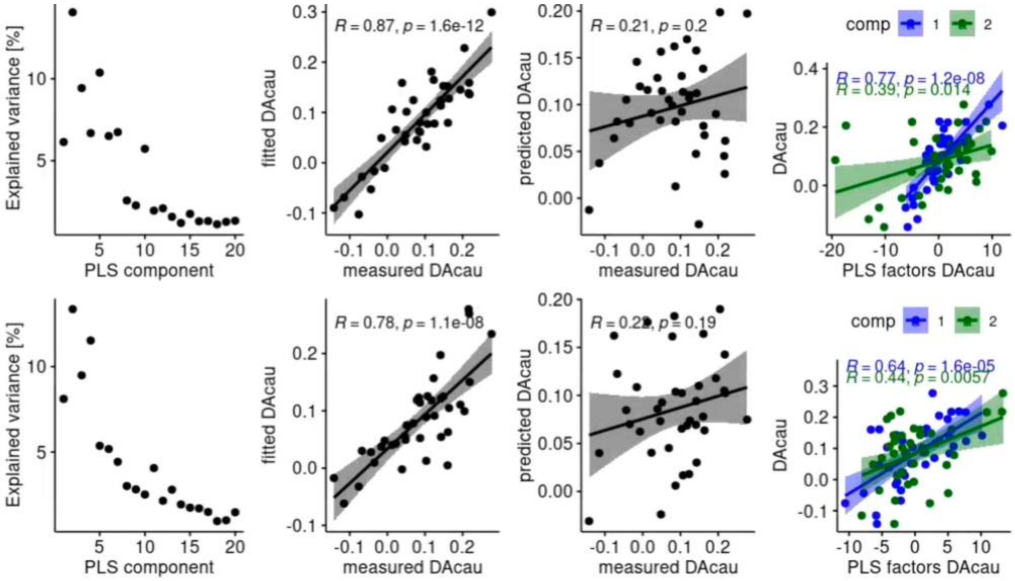

Figure S17: Partial least squares (PLS) regression predicting DA increases in caudate from the secondary gradient across 438 brain parcels. The top row includes: (i) explained variance by PLS components, (ii) fitted values from leave-one-out cross-validation (LOO-CV) versus measured values for the PL session, (iii) predicted D1 receptor availability in MP using the PLS model trained on PL, and (iv) the relationship between the measured metric and PLS factors for components 1 and 2. The bottom row shows the same metrics but with the model trained on MP and tested on PL

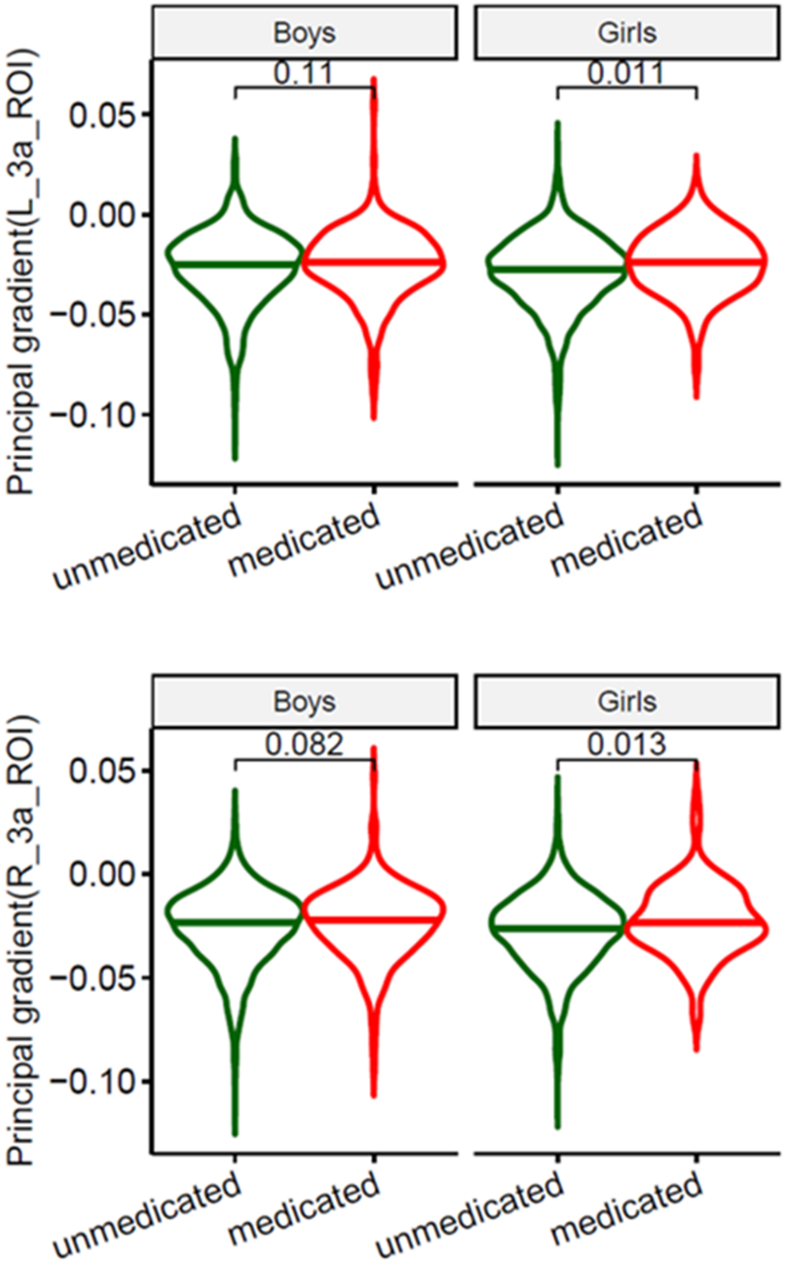

Figure S18. Stimulant-related gradient compression in ABCD boys and girls. Violin plots illustrate significantly higher gradient values in bilateral anterior primary somatomotor cortex (area 3a) for children treated with stimulant medications (methylphenidate or amphetamines; 105 girls, 274 boys) compared to unmedicated peers (2,260 girls, 2,319 boys), independently for boys and girls, after correcting for inattention, sex, age, head motion, scanner type, and research site; P-values are from two-sided two-sample t-test.

Supplementary Table S1: Summary of primary and secondary gradient strengths for placebo and methylphenidate conditions across iCSC438 parcels showing significant effects of 60 mg oral methylphenidate in 38 healthy adults. Mean values of the primary and secondary gradient strengths derived from functional connectivity data for each iCSC438 parcel that exhibited significant differences between methylphenidate (MP) and placebo (PL) conditions ($p < 0.05$, FDR-corrected).

**Brain region**	**Primary (PL)**	**Primary (MP)**	**MP>PL (t)**	**Secondary (PL)**	**Secondary (MP)**	**MP>PL (t)**
**R_V1 - Right primary visual cortex**	0.0002	-0.0065		0.0498	0.0673	3.1
**R_V6 - Right V6 (visual area)**	-0.0261	-0.0116	3.9	0.0258	0.0272	
**R_4 - Right area 4 (motor cortex)**	-0.0525	-0.0266	6.4	-0.0220	-0.0298	
**R_3b - Right somatosensory cortex, area 3b**	-0.0456	-0.0198	7.1	-0.0197	-0.0223	
**R_POS2 - Right Parieto-Occipital Sulcus Area 2**	0.0197	0.0096	-3.5	-0.0122	-0.0053	
**R_IPS1 - Right intraparietal sulcus, area 1**	-0.0028	-0.0068		0.0589	0.0447	-3.5
**R_A1 - Right primary auditory cortex**	-0.0298	-0.0181	4.0	-0.0181	-0.0125	
**R_PSL - Right PeriSylvian Language Area**	-0.0096	-0.0189	-2.8	-0.0044	0.0013	
**R_5m - Right area 5m (somatosensory)**	-0.0375	-0.0106	7.0	-0.0143	-0.0157	
**R_5mv - Right area 5mv (visual)**	-0.0399	-0.0256	4.2	-0.0113	-0.0145	
**R_5L - Right area 5L (somatosensory)**	-0.0333	-0.0135	5.6	-0.0151	-0.0154	
**R_24dd - Right area 24dd (cingulate)**	-0.0531	-0.0279	6.9	-0.0252	-0.0303	
**R_24dv - Right area 24dv (cingulate)**	-0.0385	-0.0227	5.1	-0.0233	-0.0208	
**R_7AL - Right lateral area 7 (parietal)**	-0.0319	-0.0217	3.0	-0.0044	-0.0088	
**R_VIP - Right Ventral IntraParietal Complex**	-0.0149	-0.0106		0.0368	0.0235	-2.7
**R_1 - Right area 1 (somatosensory)**	-0.0460	-0.0199	6.4	-0.0142	-0.0233	
**R_2 - Right area 2 (somatosensory)**	-0.0437	-0.0255	5.2	-0.0073	-0.0144	
**R_3a - Right area 3a (somatosensory)**	-0.0516	-0.0262	6.8	-0.0188	-0.0224	
**R_6d - Right area 6d (premotor)**	-0.0384	-0.0234	4.4	-0.0241	-0.0268	
**R_6mp - Right area 6mp (premotor)**	-0.0443	-0.0256	5.1	-0.0259	-0.0291	
**R_p32pr - Right area p32pr (parietal)**	-0.0185	-0.0275	-2.7	-0.0271	-0.0246	
**R_8BM - Right area 8BM (frontal)**	0.0212	0.0120	-3.0	-0.0314	-0.0277	
**R_IFJa - Right inferior frontal junction area (anterior)**	0.0041	-0.0037	-2.7	-0.0054	-0.0029	
**R_IFSp - Right inferior frontal sulcus, posterior**	0.0117	0.0024	-3.2	-0.0083	-0.0089	
**R_IFSa - Right inferior frontal sulcus, anterior**	0.0118	-0.0037	-5.0	-0.0142	-0.0150	
**R_46 - Right area 46 (prefrontal)**	0.0172	0.0036	-3.8	-0.0293	-0.0268	
**R_a9-46v - Right area A9-46v (ventral prefrontal)**	0.0236	0.0134	-3.2	-0.0241	-0.0206	
**R_9-46d - Right area 9-46d (dorsal prefrontal)**	0.0197	0.0048	-4.0	-0.0326	-0.0283	
**R_43 - Right area 43 (auditory)**	-0.0368	-0.0272	2.8	-0.0273	-0.0258	
**R_OP4 - Right Opercular area 4**	-0.0459	-0.0284	4.8	-0.0228	-0.0218	
**R_OP1 - Right Opercular area 1**	-0.0444	-0.0243	6.1	-0.0189	-0.0188	
**R_OP2-3 - Right Opercular area 2-3**	-0.0391	-0.0226	5.2	-0.0185	-0.0166	

**R_RI - Right RetroInsular Cortex**	-0.0478	-0.0265	6.0	-0.0202	-0.0115	
**R_PFcm - Right area PFcm**	-0.0391	-0.0277	3.5	-0.0174	-0.0147	
**R_TA2 - Right temporal area TA2**	-0.0182	-0.0108	3.1	-0.0185	-0.0126	
**R_FOP4 - Right Frontal OPercular Area 4**	-0.0064	-0.0159	-3.2	-0.0228	-0.0228	
**R_MI - Right Middle Insular Area**	-0.0069	-0.0170	-3.6	-0.0171	-0.0173	
**R_AVI - Right Anterior Ventral Insular Area**	0.0118	0.0037	-3.2	-0.0210	-0.0179	
**R_AAIC - Right Anterior Agranular Insula Complex**	0.0067	0.0045		-0.0160	-0.0078	2.7
**R_FOP2 - Right Area Frontal Opercular 2**	-0.0292	-0.0203	3.4	-0.0162	-0.0162	
**R_EC - Right Entorhinal Cortex**	0.0088	0.0077		-0.0045	0.0059	2.8
**R_ProS - Right ProStriate Area**	-0.0187	-0.0048	5.4	0.0168	0.0084	
**R_PeEc - Right Perirhinal Ectorhinal Cortex**	0.0068	0.0056		-0.0056	0.0070	3.0
**R_PBelt - Right ParaBelt Complex**	-0.0357	-0.0193	5.0	-0.0197	-0.0126	
**R_A5 - Right Auditory 5 Complex**	-0.0183	-0.0068	3.2	-0.0144	-0.0108	
**R_TGd - Right Area TG dorsal**	0.0107	0.0130		-0.0125	-0.0005	2.9
**R_TE1p - Right temporal area TE1p (posterior)**	0.0174	0.0079	-3.1	0.0032	0.0078	
**R_TE2a - Right temporal area TE2a (anterior)**	0.0171	0.0078	-2.8	-0.0136	0.0033	3.6
**R_TF - Right area TF**	0.0103	0.0050		0.0012	0.0170	3.4
**R_DVT - Right Dorsal Transitional Visual Area**	-0.0173	-0.0072	3.0	0.0271	0.0249	
**R_PF - Right area PF**	-0.0113	-0.0208	-2.7	-0.0151	-0.0150	
**R_PFm - Right area PFm**	0.0278	0.0189	-2.8	-0.0233	-0.0171	
**R_VMV1 - Right Ventral Medial Visual Area 1**	-0.0146	-0.0055	3.0	0.0393	0.0375	
**R_pOFC - Right posterior orbitofrontal cortex**	0.0135	0.0111		-0.0085	0.0019	2.9
**R_Ig - Right Insular Granular Complex**	-0.0421	-0.0232	6.0	-0.0215	-0.0174	
**R_p47r - Right posterior area 47r**	0.0191	0.0087	-3.5	-0.0186	-0.0181	
**R_TGv - Right Area TG Ventral**	0.0089	0.0066		-0.0121	0.0005	3.1
**R_MBelt - Right medial ParaBelt Complex**	-0.0242	-0.0169	3.1	-0.0194	-0.0152	
**R_LBelt - Right lateral ParaBelt Complex**	-0.0332	-0.0188	4.8	-0.0174	-0.0100	
**R_A4 - Right area 4 (motor cortex)**	-0.0300	-0.0181	3.4	-0.0174	-0.0157	
**R_TE1m - Right temporal area TE1m (medial)**	0.0230	0.0132	-3.0	-0.0124	-0.0039	
**R_a32pr - Right Area anterior 32 prime**	0.0066	-0.0048	-3.6	-0.0296	-0.0277	
**L_V1 - Left primary visual cortex**	-0.0015	-0.0045		0.0537	0.0704	2.9
**L_V6 - Left V6 (visual area)**	-0.0220	-0.0091	3.3	0.0268	0.0271	
**L_4 - Left area 4 (motor cortex)**	-0.0549	-0.0293	6.2	-0.0240	-0.0314	
**L_3b - Left somatosensory cortex, area 3b**	-0.0461	-0.0236	6.0	-0.0178	-0.0224	
**L_A1 - Left primary auditory cortex**	-0.0315	-0.0189	4.7	-0.0146	-0.0140	
**L_STV - Left superior temporal sulcus, ventral**	-0.0096	-0.0121		0.0065	-0.0087	-3.5
**L_POS1 - Left Parieto-Occipital Sulcus Area 1**	0.0120	0.0222	3.2	-0.0027	-0.0068	
**L_5m - Left area 5m (somatosensory)**	-0.0336	-0.0141	4.9	-0.0148	-0.0116	
**L_5mv - Left area 5mv (visual)**	-0.0372	-0.0256	2.9	-0.0146	-0.0186	

**L_5L - Left area 5L (somatosensory)**	-0.0385	-0.0199	5.1	-0.0188	-0.0191	
**L_24dd - Left area 24dd (cingulate)**	-0.0482	-0.0258	5.9	-0.0207	-0.0271	
**L_24dv - Left area 24dv (cingulate)**	-0.0377	-0.0224	5.7	-0.0229	-0.0190	
**L_7AL - Left lateral area 7 (parietal)**	-0.0305	-0.0247		0.0010	-0.0124	-3.1
**L_7Am - Left area 7Am (parietal)**	-0.0165	-0.0138		0.0057	-0.0081	-3.3
**L_7PL - Left area 7PL (parietal)**	-0.0051	-0.0066		0.0172	0.0048	-3.2
**L_1 - Left area 1 (somatosensory)**	-0.0428	-0.0223	5.1	-0.0151	-0.0230	
**L_2 - Left area 2 (somatosensory)**	-0.0445	-0.0288	4.7	-0.0088	-0.0173	
**L_3a - Left area 3a (somatosensory)**	-0.0561	-0.0271	8.0	-0.0202	-0.0229	
**L_6d - Left area 6d (premotor)**	-0.0359	-0.0251	3.1	-0.0185	-0.0260	
**L_6mp - Left area 6mp (premotor)**	-0.0418	-0.0274	4.0	-0.0221	-0.0273	
**L_6r - Left area 6r (premotor)**	-0.0069	-0.0159	-2.9	-0.0117	-0.0171	
**L_IFJp - Left inferior frontal junction area (posterior)**	0.0082	-0.0010	-3.6	-0.0049	-0.0057	
**L_IFSa - Left inferior frontal sulcus, anterior**	0.0122	0.0020	-3.6	-0.0120	-0.0132	
**L_p9-46v - Left area p9-46v (ventral prefrontal)**	0.0168	0.0071	-3.3	-0.0191	-0.0181	
**L_46 - Left area 46 (prefrontal)**	0.0098	-0.0046	-4.1	-0.0240	-0.0236	
**L_a9-46v - Left area A9-46v (ventral prefrontal)**	0.0224	0.0133	-3.0	-0.0218	-0.0211	
**L_43 - Left area 43 (auditory)**	-0.0387	-0.0275	3.0	-0.0196	-0.0240	
**L_OP4 - Left Opercular area 4**	-0.0452	-0.0293	4.8	-0.0169	-0.0223	
**L_OP1 - Left Opercular area 1**	-0.0430	-0.0254	5.8	-0.0155	-0.0183	
**L_OP2-3 - Left Opercular area 2-3**	-0.0403	-0.0236	5.7	-0.0185	-0.0172	
**L_RI - Left RetroInsular Cortex**	-0.0457	-0.0256	6.6	-0.0158	-0.0147	
**L_PFcm - Left area PFcm**	-0.0403	-0.0306	3.4	-0.0159	-0.0141	
**L_FOP4 - Left Frontal OPercular Area 4**	-0.0091	-0.0191	-3.5	-0.0201	-0.0232	
**L_MI - Left Middle Insular Area**	-0.0080	-0.0166	-3.2	-0.0191	-0.0183	
**L_AIP - Left anterior intraparietal sulcus**	-0.0078	-0.0108		0.0128	0.0011	-3.4
**L_ProS - Left ProStriate Area**	-0.0139	0.0004	5.2	0.0142	0.0087	
**L_PBelt - Left ParaBelt Complex**	-0.0357	-0.0215	4.9	-0.0129	-0.0162	
**L_TE1p - Left temporal area TE1p (posterior)**	0.0216	0.0134	-2.7	0.0044	0.0026	
**L_PHT - Left area PHT**	0.0056	-0.0034	-2.7	0.0209	0.0163	
**L_PF - Left area PF**	-0.0080	-0.0220	-4.3	-0.0097	-0.0161	
**L_VMV1 - Left Ventral Medial Visual Area 1**	-0.0181	-0.0043	4.8	0.0329	0.0290	
**L_Ig - Left Insular Granular Complex**	-0.0376	-0.0232	5.0	-0.0153	-0.0181	
**L_MBelt - Left medial ParaBelt Complex**	-0.0243	-0.0180	2.8	-0.0159	-0.0163	
**L_LBelt - Left lateral ParaBelt Complex**	-0.0345	-0.0211	4.3	-0.0135	-0.0154	
**L_A4 - Left area 4 (motor cortex)**	-0.0340	-0.0219	3.5	-0.0113	-0.0168	
**THA-VPm-rh - Right Thalamus, Ventral Posterior Medial nucleus**	0.0030	-0.0052	-4.6	-0.0068	-0.0041	
**THA-VAi-rh - Right Thalamus, Ventral Anterior Intermediate nucleus**	0.0130	-0.0021	-6.3	-0.0156	-0.0123	

**THA-VAs-rh - Right Thalamus, Ventral Anterior Sensory nucleus**	0.0164	0.0022	-5.7	-0.0136	-0.0118	
**THA-DAm-rh - Right Thalamus, Dorsal Anterior Medial nucleus**	0.0144	0.0011	-5.5	-0.0120	-0.0080	
**THA-DAl-rh - Right Thalamus, Dorsal Anterior Lateral nucleus**	0.0055	-0.0059	-5.0	-0.0090	-0.0079	
**PUT-VA-rh - Right Putamen, Ventral Anterior region**	0.0055	-0.0046	-4.8	-0.0092	-0.0150	
**PUT-DA-rh - Right Putamen, Dorsal Anterior region**	0.0080	-0.0064	-7.4	-0.0119	-0.0108	
**PUT-VP-rh - Right Putamen, Ventral Posterior region**	0.0031	-0.0087	-5.3	-0.0100	-0.0076	
**PUT-DP-rh - Right Putamen, Dorsal Posterior region**	0.0056	-0.0061	-5.1	-0.0138	-0.0072	
**CAU-VA-rh - Right Caudate, Ventral Anterior region**	0.0124	0.0045	-4.1	-0.0160	-0.0139	
**CAU-DA-rh - Right Caudate, Dorsal Anterior region**	0.0165	0.0070	-4.1	-0.0191	-0.0160	
**CAU-body-rh - Right Caudate, Body region**	0.0156	0.0044	-5.2	-0.0164	-0.0145	
**CAU-tail-rh - Right Caudate, Tail region**	0.0141	0.0015	-6.5	-0.0133	-0.0095	
**aGP-rh - Right Anterior Globus Pallidus**	0.0058	0.0007	-3.0	-0.0102	-0.0071	
**THA-VPm-lh - Left Thalamus, Ventral Posterior Medial nucleus**	0.0029	-0.0038	-3.6	-0.0080	-0.0057	
**THA-VAi-lh - Left Thalamus, Ventral Anterior Intermediate nucleus**	0.0131	0.0004	-5.2	-0.0153	-0.0112	
**THA-VAs-lh - Left Thalamus, Ventral Anterior Sensory nucleus**	0.0160	0.0033	-5.1	-0.0162	-0.0117	
**THA-DAm-lh - Left Thalamus, Dorsal Anterior Medial nucleus**	0.0137	0.0037	-4.4	-0.0129	-0.0135	
**THA-DAl-lh - Left Thalamus, Dorsal Anterior Lateral nucleus**	0.0082	-0.0050	-5.6	-0.0135	-0.0106	
**PUT-VA-lh - Left Putamen, Ventral Anterior region**	0.0062	-0.0023	-3.6	-0.0137	-0.0155	
**PUT-DA-lh - Left Putamen, Dorsal Anterior region**	0.0059	-0.0052	-5.9	-0.0144	-0.0131	
**PUT-VP-lh - Left Putamen, Ventral Posterior region**	0.0014	-0.0090	-4.6	-0.0136	-0.0135	
**PUT-DP-lh - Left Putamen, Dorsal Posterior region**	0.0042	-0.0052	-4.2	-0.0142	-0.0109	
**CAU-body-lh - Left Caudate, Body region**	0.0154	0.0095	-2.7	-0.0135	-0.0134	
**CAU-tail-lh - Left Caudate, Tail region**	0.0132	0.0060	-3.5	-0.0111	-0.0073	
**aGP-lh - Left Anterior Globus Pallidus**	0.0059	0.0015	-2.7	-0.0080	-0.0072	
**Left_V - Left Cerebellar Lobe, Region V (anterior lobe)**	0.0034	-0.0061	-3.3	-0.0032	0.0002	
**Right_V - Right Cerebellar Lobe, Region V (anterior lobe)**	0.0033	-0.0050	-2.9	0.0005	0.0015	
**Left_VI - Left Cerebellar Lobe, Region VI (anterior lobe)**	0.0096	-0.0067	-4.7	0.0046	0.0089	
**Right_VI - Right Cerebellar Lobe, Region VI (anterior lobe)**	0.0093	-0.0022	-3.4	0.0080	0.0107	
**Vermis_CrusI - Vermis, Crus I (part of the posterior lobe)**	0.0040	0.0013	-3.0	-0.0018	-0.0017	
**Vermis_CrusII - Vermis, Crus II (part of the posterior lobe)**	0.0082	-0.0002	-3.4	0.0188	0.0188	
**Left_VIIb - Left Cerebellar Lobe, Region VIIb (posterior lobe, cerebellar hemisphere)**	0.0110	-0.0001	-3.6	0.0262	0.0363	
**Right_VIIb - Right Cerebellar Lobe, Region VIIb (posterior lobe, cerebellar hemisphere)**	0.0113	-0.0015	-4.3	0.0217	0.0287	

**Left_VIIIa - Left Cerebellar Lobe, Region VIIIa (posterior lobe)**	0.0046	-0.0045	-3.0	0.0241	0.0292	
**Right_VIIIa - Right Cerebellar Lobe, Region VIIIa (posterior lobe)**	0.0058	-0.0054	-3.6	0.0233	0.0262	
**Left_VIIIb - Left Cerebellar Lobe, Region VIIIb (posterior lobe)**	0.0066	-0.0035	-3.6	0.0170	0.0254	
**Right_VIIIb - Right Cerebellar Lobe, Region VIIIb (posterior lobe)**	0.0060	-0.0037	-3.4	0.0202	0.0241	
**Vermis_IX - Vermis, Region IX (part of the posterior lobe, near the nodulus)**	0.0100	0.0032	-2.9	0.0122	0.0174	
**Left_X - Left Cerebellar Lobe, Region X (flocculonodular lobe)**	0.0100	-0.0004	-4.6	0.0146	0.0172	
**Vermis_X - Vermis, Region X (flocculonodular lobe)**	0.0088	0.0026	-3.9	0.0030	0.0035	
**Right_X - Right Cerebellar Lobe, Region X (flocculonodular lobe)**	0.0090	0.0006	-3.7	0.0188	0.0179	
