# Supplementary material for: Methylphenidate reorganizes cortical hierarchy through dopaminergic modulation
Source: Nat Commun. 2025 Dec 13;17:791. doi: 10.1038/s41467-025-67477-y (PMC12824357; doi:10.1038/s41467-025-67477-y)
Supplement: Supplementary file 2 — Reporting Summary [file 41467_2025_67477_MOESM2_ESM.pdf]

nature portfolio

Corresponding author(s): Dardo Tomasi

Last updated by author(s): Oct 23, 2025

Reporting Summary

Nature Portfolio wishes to improve the reproducibility of the work that we publish. This form provides structure for consistency and transparency in reporting. For further information on Nature Portfolio policies, see our Editorial Policies and the Editorial Policy Checklist.

Statistics

For all statistical analyses, confirm that the following items are present in the figure legend, table legend, main text, or Methods section.

n/a	Confirmed	
☐	☒	The exact sample size (*n*) for each experimental group/condition, given as a discrete number and unit of measurement
☐	☒	A statement on whether measurements were taken from distinct samples or whether the same sample was measured repeatedly
☐	☒	The statistical test(s) used AND whether they are one- or two-sided<br>*Only common tests should be described solely by name; describe more complex techniques in the Methods section.*
☐	☒	A description of all covariates tested
☐	☒	A description of any assumptions or corrections, such as tests of normality and adjustment for multiple comparisons
☐	☒	A full description of the statistical parameters including central tendency (e.g. means) or other basic estimates (e.g. regression coefficient) AND variation (e.g. standard deviation) or associated estimates of uncertainty (e.g. confidence intervals)
☐	☒	For null hypothesis testing, the test statistic (e.g. *F*, *t*, *r*) with confidence intervals, effect sizes, degrees of freedom and *P* value noted<br>*Give P values as exact values whenever suitable.*
☒	☐	For Bayesian analysis, information on the choice of priors and Markov chain Monte Carlo settings
☒	☐	For hierarchical and complex designs, identification of the appropriate level for tests and full reporting of outcomes
☐	☒	Estimates of effect sizes (e.g. Cohen's *d*, Pearson's *r*), indicating how they were calculated

Our web collection on statistics for biologists contains articles on many of the points above.

Software and code

Policy information about availability of computer code

Data collection	The data in this study were collected using a combination of commercial, open-source, and custom software. MRI data were acquired on a Siemens 3T Prisma scanner using proprietary Siemens syngo MR VD13D software. PET data were collected on a Siemens Biograph mMR system using Siemens syngo PET Acquisition and Reconstruction software. Task stimuli for the visual attention task were presented using Visual Basic 6.0 with synchronization to scanner pulses.
Data analysis	MATLAB R2022a; R4.0; RStudio 2023.03.0

For manuscripts utilizing custom algorithms or software that are central to the research but not yet described in published literature, software must be made available to editors and reviewers. We strongly encourage code deposition in a community repository (e.g. GitHub). See the Nature Portfolio guidelines for submitting code & software for further information.

Data

Policy information about availability of data

All manuscripts must include a data availability statement. This statement should provide the following information, where applicable:

- Accession codes, unique identifiers, or web links for publicly available datasets
- A description of any restrictions on data availability
- For clinical datasets or third party data, please ensure that the statement adheres to our policy

The deidentified summary data generated in this study will be deposited in the Open Science Framework (OSF) repository and made available for review.

Deidentified individual-level data can be obtained from the corresponding author upon reasonable request. In accordance with NIH policy and institutional requirements, data sharing involving human subjects may require Data Transfer Agreements (DTAs) between institutions to ensure compliance with privacy, legal, and ethical standards. As such, the data cannot be made publicly available but will be shared upon request through appropriate channels.

Research involving human participants, their data, or biological material

Policy information about studies with human participants or human data. See also policy information about sex, gender (identity/presentation), and sexual orientation and race, ethnicity and racism.

Reporting on sex and gender	This study included both male and female participants, and sex was recorded as a demographic variable at enrollment. While the primary analyses were not stratified by sex due to sample size and study objectives, sex was included as a covariate in relevant statistical models to account for potential effects. Gender identity was not separately assessed. However, in the external validation analysis using the ABCD dataset, we examined stimulant-related effects separately in boys and girls. Future studies with larger and more balanced samples may be warranted to further explore potential sex- and gender-related differences in greater detail.
Reporting on race, ethnicity, or other socially relevant groupings	Race and ethnicity were self-reported by participants at enrollment and documented in accordance with NIH guidelines. These variables were collected to characterize the sample and to account for potential confounding effects in the analyses. Although the study did not focus on race- or ethnicity-based comparisons, race was included as a covariate in statistical models to adjust for its potential influence on neuroimaging and behavioral outcomes. We recognize the importance of social and structural determinants of health in neuroscience research and encourage future studies with larger and more diverse samples to explore these factors more directly.
Population characteristics	Participants in this study were healthy adult volunteers recruited from the local community. Eligibility criteria included ages 18–50, right-handedness, and no history of neurological or psychiatric disorders, substance use disorders, or current use of psychoactive medications. All participants had normal or corrected-to-normal vision and passed a medical screening that included physical examination, urine toxicology, and MRI safety assessment. The final discovery cohort included 38 individuals (21 female), and a separate replication cohort included 20 individuals (10 female). Demographic variables such as age, sex, and race were recorded, and race was included as a covariate in statistical analyses to account for potential confounding effects.
Recruitment	Participants were recruited through flyers, online advertisements, and word-of-mouth in the greater Washington, D.C. area. Recruitment targeted healthy adults with no history of neurological, psychiatric, or substance use disorders. Interested individuals completed an initial phone screening, followed by an in-person clinical evaluation to confirm eligibility. This evaluation included a structured medical and psychiatric interview, urine toxicology screening, and MRI safety assessment. Written informed consent was obtained from all participants prior to enrollment in accordance with the guidelines of the NIH Institutional Review Board.
Ethics oversight	This study was conducted in accordance with the Declaration of Helsinki and approved by the Institutional Review Board (IRB) of National Institutes of Health. All participants provided written informed consent prior to participation. Procedures involving human subjects, including data collection, storage, and analysis, adhered to institutional and federal guidelines to ensure participant confidentiality and safety. Any modifications to the protocol were reviewed and approved by the IRB.

Note that full information on the approval of the study protocol must also be provided in the manuscript.

Field-specific reporting

Please select the one below that is the best fit for your research. If you are not sure, read the appropriate sections before making your selection.

☒ Life sciences ☐ Behavioural & social sciences ☐ Ecological, evolutionary & environmental sciences

For a reference copy of the document with all sections, see nature.com/documents/nr-reporting-summary-flat.pdf

Life sciences study design

All studies must disclose on these points even when the disclosure is negative.

Sample size	Sample size determination was guided by prior studies examining the effects of methylphenidate on brain function and by practical constraints related to PET/MRI data acquisition. Power analyses were not feasible due to the lack of directly comparable multimodal imaging studies; instead, we followed precedent from similar neuroimaging research using within-subject designs. The discovery cohort (n=38) was selected to detect robust within-subject effects of methylphenidate on functional gradients and dopamine signaling. The independent replication cohort (n=20) was enrolled to assess the reproducibility of key findings. This sample size is consistent with previous PET and fMRI studies investigating pharmacological effects on brain function.
Data exclusions	Participants were excluded from analysis if they did not complete both imaging sessions, exhibited excessive head motion during fMRI (defined as >2 mm translation or >2° rotation), or if PET data were incomplete or of poor quality due to scanner or injection issues.
Replication	A replication cohort was analyzed using the same protocols to confirm the robustness of the findings.
Randomization	Participants underwent a randomized, double-blind, placebo-controlled crossover design. The order of drug administration (methylphenidate or placebo) was randomly assigned using a computer-generated randomization schedule to minimize potential order effects.

Blinding	Both participants and researchers conducting assessments were blinded to condition assignment to reduce bias. Randomization was stratified to ensure balanced representation across sex and age ranges.

Reporting for specific materials, systems and methods

We require information from authors about some types of materials, experimental systems and methods used in many studies. Here, indicate whether each material, system or method listed is relevant to your study. If you are not sure if a list item applies to your research, read the appropriate section before selecting a response.

Materials & experimental systems

n/a	Involved in the study	
☒	☐	Antibodies
☒	☐	Eukaryotic cell lines
☒	☐	Palaeontology and archaeology
☒	☐	Animals and other organisms
☐	☒	Clinical data
☒	☐	Dual use research of concern
☒	☐	Plants

Methods

n/a	Involved in the study	
☒	☐	ChIP-seq
☒	☐	Flow cytometry
☐	☒	MRI-based neuroimaging

Clinical data

Policy information about clinical studies

All manuscripts should comply with the ICMJE guidelines for publication of clinical research and a completed CONSORT checklist must be included with all submissions.

Clinical trial registration	NCT03190954<br>NCT03326245
Study protocol	https://clinicaltrials.gov/
Data collection	NIH
Outcomes	The primary outcomes of the study included changes in functional brain gradients derived from resting-state fMRI and task-based attention performance following administration of methylphenidate versus placebo. Secondary outcomes included associations between these functional brain changes and dopamine receptor availability measured via PET imaging.

Plants

Seed stocks	n/a
Novel plant genotypes	n/a
Authentication	n/a

Magnetic resonance imaging

Experimental design

Design type	resting-state
Design specifications	eyes open
Behavioral performance measures	Behavioral performance was assessed using a sustained attention task with varying cognitive load conditions (2-back and 3-back tasks). Key performance metrics included accuracy (percentage of correct responses) and reaction time (RT) for correct trials. These measures were collected during both placebo and methylphenidate sessions and served to quantify individual differences in attentional performance and drug response. Behavioral data were analyzed to examine the effects of methylphenidate, load condition, and their interaction, as well as to explore associations with imaging-based outcomes.

Acquisition

Imaging type(s)	functional and structural
Field strength	3T
Sequence & imaging parameters	MRI data were acquired using a Siemens 3T Prisma scanner with a 32-channel head coil. Functional MRI (fMRI) data were collected using a T2*-weighted single- and multi-echo multiband echo-planar imaging sequences. Structural images were acquired using a T1-weighted magnetization-prepared rapid gradient-echo (MPRAGE) sequence. Resting-state and task-based fMRI scans were obtained in both placebo and methylphenidate sessions. PET data were collected using a Siemens HRRT, PET-CT, or Biograph mMR scanners with [^{11}C]raclopride.
Area of acquisition	Brain

Diffusion MRI ☐ Used ☒ Not used

Preprocessing

Preprocessing software	All MRI data were preprocessed using the fMRIPrep pipeline, which provides standardized anatomical and functional preprocessing. T1-weighted images underwent intensity non-uniformity correction, skull stripping, segmentation, and spatial normalization to MNI152NLin2009cAsym space. Functional MRI preprocessing included slice-timing correction, motion correction, susceptibility distortion correction, co-registration to T1-weighted space, and resampling to standard and native spaces. Surface-based processing was performed in CIFTI grayordinate space using FreeSurfer. Confound regressors, including motion parameters and aCompCor components, were estimated and used in subsequent nuisance regression. PET data were processed using the MAGIA pipeline, which enables automated motion correction, co-registration to structural MRI, and kinetic modeling. Regional binding potential values were estimated using the simplified reference tissue model (SRTM), with cerebellar gray matter as the reference region. To account for scanner-related variability across the HRRT and PET/CT systems, we applied ComBat harmonization to regional PET outcomes.
Normalization	PET data were standardized using the MAGIA pipeline, which included normalization of tracer uptake values to a reference region to account for individual variability in signal intensity. Additionally, to harmonize PET data acquired on different scanner platforms (HRRT and PET-CT), we applied the ComBat method to adjust for scanner-related effects while preserving biological variability.
Normalization template	MNI152
Noise and artifact removal	To minimize the impact of noise and artifacts on the imaging data, rigorous preprocessing steps were implemented. For fMRI data, the fMRIPrep pipeline was used, which incorporates motion correction, susceptibility distortion correction, and physiological noise regression, including removal of signals from white matter and cerebrospinal fluid compartments. Additionally, framewise displacement metrics were calculated to identify and censor volumes with excessive motion. For PET data, the MAGIA pipeline included corrections for scatter, attenuation, and motion artifacts. Scanner-specific noise and batch effects were further addressed through the application of the ComBat harmonization method, which adjusts for systematic variability while preserving biologically relevant signal. Together, these procedures ensured high-quality, artifact-reduced datasets suitable for robust statistical analysis.
Volume censoring	Framewise displacement (FD) and the root mean square (RMS) of voxelwise intensity differences between successive time points (e.g., DVAR) were computed, and treated as nuisance regressors, and volumes exceeding 0.5 mm FD and 0.5% DVARS were flagged to facilitate posterior denoising based on scrubbing.

Statistical modeling & inference

Model type and settings	Statistical inference was performed using linear mixed-effects (LME) models implemented in Matlab to evaluate the effect of methylphenidate (MP) versus placebo (PL) on functional connectivity gradient strength. Separate models were fit for each gradient, with drug condition as a fixed effect and age, sex, race, BMI, and IQ included as covariates to control for potential confounding factors. A random intercept was specified for each participant to account for within-subject dependencies across multiple resting-state scans collected in randomized order under each drug condition. Model selection and inference were conducted using likelihood ratio tests and restricted maximum likelihood estimation. To correct for multiple comparisons, false discovery rate (FDR) correction was applied. Additionally, spatial correlations between MP-induced gradient shifts and PET-derived maps of dopamine and norepinephrine transporter densities were assessed using spin tests to account for spatial autocorrelation, with significance determined by comparison to null distributions generated through 10,000 random permutations.
Effect(s) tested	The primary effect tested was the impact of methylphenidate administration on functional connectivity gradient strength, controlling for demographic covariates.

Specify type of analysis: ☐ Whole brain ☐ ROI-based ☒ Both

Anatomical location(s): 91282 grayordinates and 438 ROIs covering the whole brain

Statistic type for inference (See Eklund et al. 2016)	vertex-wise and ROI-wise

Correction | FDR

Models & analysis

n/a	Involved in the study
☐	☒ Functional and/or effective connectivity
☒	☐ Graph analysis
☐	☒ Multivariate modeling or predictive analysis

Functional and/or effective connectivity: n/a

Multivariate modeling and predictive analysis: Partial Least Squares (PLS) regression was conducted in R using the pls package to predict dopamine-related metrics (D1 receptor availability, D2 receptor availability, and methylphenidate-induced dopamine increases) from low-dimensional functional connectivity gradient components. Both predictor (gradient components) and outcome variables were standardized via z-scoring to ensure comparability. The optimal number of PLS components was selected through cross-validation by identifying the model that minimized prediction error. Model performance was assessed using R^2 and root mean squared error (RMSE). To support statistical inference, permutation testing was applied to evaluate the significance of the predictive relationships, controlling for multiple comparisons with false discovery rate correction. This approach ensured robust assessment of multivariate associations between brain connectivity gradients and neurochemical measures.
